# The risk of over-interpreting in silico predictions as clinical evidence: a comment on Roy et al. (2024)

**DOI:** 10.1186/s44342-026-00082-7

**Published:** 2026-10-08

**Authors:** Dimitri Tchernitchko

**Affiliations:** Department of Genetics, Bichat Hospital (APHP), University of Paris Cité, Paris, France

**Keywords:** CYLD, Missense variants, Variant interpretation, In silico prediction, ACMG/AMP, ClinVar

## Abstract

Roy et al. reported an extensive computational analysis of nonsynonymous single-nucleotide variants (nsSNPs) in the human *CYLD* gene and characterized several variants as potentially disease- or cancer-associated. Although such analyses can be valuable for generating hypotheses, computational predictions should be clearly distinguished from clinical or functional evidence. Among the 18 “high-risk nsSNPs” prioritized by the authors, only p.Glu747Gly (E747G) has established clinical evidence supporting pathogenicity, whereas the remaining variants lack sufficient evidence for pathogenic classification and should be considered variants of uncertain significance pending further evidence. We highlight three major concerns: the lack of independence among some prediction tools used for variant prioritization; the interpretation of somatic mutation clustering as evidence for germline disease association; and the presentation of variants with no established clinical or experimental evidence as disease-associated. Under the ACMG/AMP framework, computational evidence can provide supporting evidence but cannot substitute for clinical, population, segregation, or functional evidence. We suggest that the terminology and conclusions of the original article be revised to distinguish computationally predicted effects from demonstrated disease associations.

## Main text

Roy et al. [1] recently presented an extensive computational analysis of nonsynonymous single-nucleotide variants (nsSNPs) in the human *CYLD* gene. Although such in silico approaches can generate useful hypotheses concerning the potential effects of variants, we have concerns regarding the clinical framing of their results, particularly the characterization of computationally predicted variants as “disease-associated” or “cancer-associated.”

First, computational prediction should be clearly distinguished from clinical evidence. Roy et al. screened 446 *CYLD* nsSNPs from dbSNP and identified 18 variants predicted to be deleterious by seven computational tools. Importantly, p.Glu747Gly (E747G; rs121908389) is a clinically established pathogenic *CYLD* variant, but its pathogenicity is not established by the computational analyses presented in this study. Hu et al. [2] identified E747G in a four-generation family with Brooke–Spiegler syndrome and demonstrated complete co-segregation with the disease phenotype. In contrast, Supplementary Table S1 of Roy et al. lists p.Leu781Pro (L781P; rs886040889) as associated with Brooke–Spiegler syndrome according to ClinVar. However, L781P is currently classified as a variant of uncertain significance (VUS) in ClinVar and should therefore not be presented as an established disease-causing variant on the basis of this database entry. More generally, concordant computational predictions cannot by themselves establish that a variant is disease-associated.

Second, the seven computational predictors do not represent seven independent lines of evidence. Of particular relevance, PredictSNP is a consensus predictor incorporating several other approaches, including PANTHER, PhD-SNP, PolyPhen-2, and SIFT. Roy et al. explicitly describe PredictSNP as a consensus program that integrates results from multiple prediction tools. Consequently, the observation that a variant is predicted to be deleterious by all seven tools should not be interpreted as seven independent pieces of evidence. The apparent concordance is partly dependent on overlapping algorithms and underlying information. This issue is particularly important when such concordance is subsequently used to support claims of disease association.

Third, the interpretation of Mutation 3D warrants clarification. Roy et al. state that Mutation 3D “verified” that mutations at H827 and R830 have a strong association with cancer. However, Mutation 3D identifies spatial clusters of mutations derived from cancer sequencing data. Such clustering of somatic cancer mutations does not establish that a specific variant is a pathogenic germline variant or that it confers hereditary cancer susceptibility. This distinction is particularly important for H827R and R830K, which were subsequently selected as the principal variants for molecular docking and molecular dynamics analyses. The authors report differences in RMSD, RMSF, radius of gyration, and hydrogen-bond formation between these mutants and wild-type CYLD, but these observations remain computational predictions of structural or interaction effects.

The structural analyses also illustrate why these findings should be regarded as hypotheses rather than convergent evidence of pathogenicity. Multiple computational approaches were used to characterize the selected variants, including conservation, stability, structural modelling, docking, and molecular dynamics. However, agreement among computational analyses does not constitute independent clinical or experimental validation.

Importantly, Roy et al. themselves acknowledge in their conclusion that their findings require in vitro and in vivo experimental validation. We agree with this qualification, but believe that it should be reflected consistently throughout the manuscript, particularly in the terminology used in the title, abstract, results, and conclusions.

Under the ACMG/AMP framework [3], computational evidence may contribute to variant interpretation as supporting evidence when appropriately applied, but it cannot independently establish pathogenicity. Clinical classification requires integration of relevant population, clinical, segregation, functional, and other evidence. Accordingly, the variants prioritized by Roy et al. should be regarded as computational candidates for further investigation, rather than established disease-associated variants, in the absence of appropriate clinical or functional evidence.

We therefore suggest that the published record be clarified by: (i) distinguishing predicted structural or functional effects from demonstrated disease associations; (ii) correcting or appropriately qualifying the ClinVar interpretation of L781P; (iii) clarifying that Mutation 3D provides information on mutation clustering in cancer datasets rather than evidence of pathogenic germline variants; and (iv) explicitly stating that the reported computational findings should not be used in isolation for clinical variant classification.

In summary, the computational workflow reported by Roy et al. may be useful for prioritizing *CYLD* variants and generating experimentally testable hypotheses. However, extensive in silico analyses should not be conflated with independent clinical or functional evidence. E747G illustrates why this distinction matters: its pathogenicity is supported by a specific disease phenotype and familial co-segregation, rather than by computational prediction alone. We believe that a clearer distinction between predicted effects and demonstrated disease associations would substantially improve the clinical interpretation and usefulness of the study. This distinction is particularly important in the era of panel-based sequencing, whole-exome sequencing, and whole-genome sequencing, which routinely identify large numbers of incidental rare variants. Erroneous interpretation based primarily on computational predictions may lead to inappropriate variant classification and, consequently, potentially harmful clinical management and genetic counselling.

## Data Availability

No datasets were generated or analysed during the current study.
